# The Interaction of Influenza A NS1 and Cellular TRBP Protein Modulates the Function of RNA Interference Machinery

**DOI:** 10.3389/fmicb.2022.859420

**Published:** 2022-04-26

**Authors:** Qi Wang, Jiaxin Wang, Yan Xu, Zhe Li, Binbin Wang, Yang Li

**Affiliations:** ^1^State Key Laboratory of Genetic Engineering, School of Life Sciences, Fudan University, Shanghai, China; ^2^CAS Key Laboratory of Animal Ecology and Conservation Biology, Institute of Zoology, Chinese Academy of Sciences, Beijing, China

**Keywords:** influenza A virus, non-structural protein 1 of IAV, TRBP, antiviral RNAi response, RNA interference machinery

## Abstract

Influenza A virus (IAV), one of the most prevalent respiratory diseases, causes pandemics around the world. The multifunctional non-structural protein 1 (NS1) of IAV is a viral antagonist that suppresses host antiviral response. However, the mechanism by which NS1 modulates the RNA interference (RNAi) pathway remains unclear. Here, we identified interactions between NS1 proteins of Influenza A/PR8/34 (H1N1; IAV-PR8) and Influenza A/WSN/1/33 (H1N1; IAV-WSN) and Dicer’s cofactor TAR-RNA binding protein (TRBP). We found that the N-terminal RNA binding domain (RBD) of NS1 and the first two domains of TRBP protein mediated this interaction. Furthermore, two amino acid residues (Arg at position 38 and Lys at position 41) in NS1 were essential for the interaction. We generated TRBP knockout cells and found that NS1 instead of NS1 mutants (two-point mutations within NS1, R38A/K41A) inhibited the process of microRNA (miRNA) maturation by binding with TRBP. PR8-infected cells showed masking of short hairpin RNA (shRNA)-mediated RNAi, which was not observed after mutant virus-containing NS1 mutation (R38A/K41A, termed PR8/3841) infection. Moreover, abundant viral small interfering RNAs (vsiRNAs) were detected *in vitro* and *in vivo* upon PR8/3841 infection. We identify, for the first time, the interaction between NS1 and TRBP that affects host RNAi machinery.

## Introduction

Influenza A viruses (IAVs) are widespread pathogens causing severe respiratory disease around the world (Neumann et al., 2010; Su et al., 2015; Coughlan and Palese, 2018). Seasonal epidemics of influenza affect 5%–15% of the global population, and cause about 250,000 to 500,000 respiratory deaths annually, with influenza A causing considerable morbidity and mortality (Li et al., 2018; Yassine et al., 2018). IAVs are negative-stranded RNA viruses belonging to the *Orthomyxoviridae* family, which contain 8 segments encoding approximately 14 proteins (Krammer et al., 2018; Ampomah and Lim, 2020). Different subtypes of IAVs are classified according to two glycoproteins, haemagglutinin (HA) and neuraminidase (NA). The three polymerase proteins (PA, PB1, and PB2) form a viral ribonucleoprotein (vRNP) with the nucleoprotein (NP)-encapsidated RNA segment in the replication of IAVs.

Non-structural protein 1 (NS1) consists of 215 to 237 amino acids (aa) and consists of an N-terminal RNA binding domain (RBD; 1 to 73 aa) and a C-terminal effector domain (ED; 85 aa to end) and joined by a linker domain (LD; 74 to 84 aa; Hale et al., 2008; Krug, 2015). The RBD non-specifically binds double-stranded RNA (dsRNA) of different lengths and mediates several interactions (Chien et al., 2004; Hale et al., 2008). Highly expressed NS1 performs a range of activities to inhibit the host antiviral response by interacting with interferon (IFN)-induced proteins and antagonizing IFN production during infection (Krug et al., 2003; Krug, 2015). Two residues of NS1, arginine 38 (R38) and lysine 41 (K41), are the key functional sites for binding dsRNA and retinoic acid-inducible gene I (RIG-I) to inhibit signal transduction (Qian et al., 1994; Wang et al., 1999; Talon et al., 2000; Wang et al., 2000; Mibayashi et al., 2007). NS1 competes with 2′-5′-oligoadenylate synthetase (2′-5’-OAS) for interaction with dsRNA, thereby blocking cleavage by RNase L of viral and cellular mRNA (Ji-Young Min, 2006). The ED also mediates functional interactions with host proteins. The interaction between NS1 and ubiquitin ligase TRIM25 suppresses RIG-I signal transduction, which requires E96/E97 residues in the ED of NS1 (Gack et al., 2009). NS1 binds to protein kinase R (PKR) at residues 123–127, which in turn inhibits PKR-mediated eukaryotic translation initiation factor eIF2α phosphorylation (Li et al., 2006; Min et al., 2007; Schierhorn et al., 2017). Furthermore, the cellular cleavage and polyadenylation specific factor 30 (CPSF30) binds to the ED, in turn blocking the maturation of pre-mRNA (Nemeroff et al., 1998).

RNA interference (RNAi) has been recognized as an important gene silencing mechanism in mammals (Ding, 2010; Guo et al., 2019). In the processing of RNAi, the stem-loop structure of precursor microRNAs (pre-miRNAs) or viral double-stranded RNA replicative intermediates (dsRNA-vRIs) are cleaved into ~22 nucleotide (nt) miRNAs or viral small interfering RNAs (vsiRNAs) by Dicer, an enzyme belonging to the RNase III family (Bernstein et al., 2001; Hutvágner et al., 2001). Then, these small RNAs (sRNAs) are loaded into Argonaute-2 (AGO2) protein, an important component of RNA-induced silencing complex (RISC), leading to silencing or degradation of target sequences (Jinek and Doudna, 2009; Ding et al., 2018). As another essential member of RISC, TRBP serves as a cofactor of Dicer in the processing of miRNAs (Chendrimada et al., 2005; Haase et al., 2005; Takahashi et al., 2018). However, whether TRBP affects miRNA abundance or isoforms remains controversial (Chendrimada et al., 2005; Haase et al., 2005; Melo et al., 2009; Lee and Doudna, 2012; Kim et al., 2014). Numerous proteins, including adenosine deaminases acting on RNA-1 (ADAR1) and the protein activator of PKR (PACT), have also been reported to enhance the cleavage activity of Dicer (Lee et al., 2006; Ota et al., 2013; Heyam et al., 2015).

Many studies indicate that host miRNA expression levels are regulated to resist viral infections. In hepatocytes, hepatitis C virus (HCV)-induced IFN-β regulates the expression of cellular miRNAs including miR-196, miR-351, and miR-431, which target the RNA genome of HCV to inhibit viral replication (Pedersen et al., 2007). Increased expression of miR-296-5p directly targets VP1 and VP3 coding sequences of the genome to inhibit the Enterovirus 71 (EV71) replication in rhabdomyosarcoma (RD) and human neuroblastoma (SKN-SH) cells (Zheng et al., 2013). In addition, miR-32 targets viral nucleic acids, which restricts the expression of primate foamy virus type 1 (PFV-1) mRNA in 293 T cells (Lecellier et al., 2005). Many viruses in turn inhibit host miRNA maturation for viral replication. Flaviviruses, including dengue viruses (DENV), Kunjin virus (KUNV), and Japanese encephalitis virus (JEV), suppress miRNA production through non-coding subgenomic flavivirus RNAs (sfRNAs) that associate with Dicer and AGO2 in infected cells (Moon et al., 2015). Human cytomegalovirus (HCMV) encodes intergenic sequences that are complementary to miR-17, resulting in its degradation (Lee et al., 2013). In addition, Zika virus (ZIKV) capsid protein binds to Dicer to dampen miRNA production in neural stem cells (NSCs; Zeng et al., 2020).

On the other hand, there is growing evidence that siRNA-based antiviral immunity plays an important role in mammals (Li et al., 2013; Maillard et al., 2013; Li et al., 2016; Qiu et al., 2017; Han et al., 2020; Zeng et al., 2020). Recent studies including ours have shown several viruses, such as Nodamura virus (NoV), IAV, Sindbis virus (SINV), and ZIKV induce vsiRNA production *in vitro* or *in vivo* (Li et al., 2013; Li et al., 2016; Zhang et al., 2020; Zhang et al., 2021). To counter the defense mechanism, many viruses encode viral suppressors of RNAi (VSRs), including NoV B2, IAV NS1, Ebolavirus (EBOV) VP35, and human enterovirus 71 (HEV71) 3A (Fabozzi et al., 2011; Li et al., 2013; Li et al., 2016; Qiu et al., 2017). For instance, VP35 directly interacts with TRBP and PACT to suppress the effects of siRNAs (Fabozzi et al., 2011). 3A inhibits siRNAs production by sequestrating viral dsRNA (Qiu et al., 2017).

Similar to several viruses, IAV has also been recognized to functionally regulate cellular miRNA expression (Terrier et al., 2013; Tan et al., 2014; Jiao et al., 2019). Moreover, our previous study has shown that vsiRNAs are detected in mammalian cells only when infected with IAV lacking NS1, suggesting that NS1 blocks vsiRNA production (Li et al., 2016). Our emerging study also determines that NS1 encoded by influenza A/WSN/1/33 (WSN) interacts with AGO2, which induces nuclear import of AGO2 (Wang et al., 2020). In addition, multiple studies have observed that NS1 interacts with many host proteins including ADAR1 and PACT that participate in the RNAi process (de Chassey et al., 2013; Tawaratsumida et al., 2014).

Although IAV NS1 has been shown to inhibit the production of sRNAs, few studies have clarified the mechanism underlying suppression by NS1. Here, we show the interaction between PR8 NS1 and TRBP, which inhibits some miRNAs production. Further analysis reveals that R38 and K41 of NS1 are vital sites for this binding. Moreover, our findings provide the first evidence for the *in vivo* production canonical duplex vsiRNAs by mutant IAV virus. Our work explains the mechanism of NS1 in modulating RNA interference machinery from a new perspective.

## Materials and Methods

### Cell Culture and Viruses

Human embryonic kidney cells (293 T) were cultured in Dulbecco’s modified Eagle’s medium (DMEM; Sigma) containing 10% fetal bovine serum (FBS; Gibco) at 37°C with 5% CO_2_. Influenza A/Puerto Rico/8/34 (H1N1), designated PR8-wild type (WT), Influenza A/WSN/1/33 (H1N1), designated WSN-WT, and the mutant virus, designated PR8/3841, were gifts from Dr. A. García-Sastre.

### Plasmids and Molecular Cloning

The sequences encoding NS1, N-NS1, C-NS1, and NS1 38/41 of PR8-WT and the sequences encoding NS1, NS1 38/41 of WSN-WT were generated by reverse transcription–polymerase chain reaction (RT-PCR) and cloned into pcDNA3.1 vector digested by EcoRI and HindIII (NEB) to generate pcDNA-NS1, pcDNA-N-NS1, pcDNA-C-NS1 and pcDNA-NS1 38/41. The open reading frame (ORF) of EGFP was cloned into the EcoRI and HindIII sites of the pcDNA3.1 vector to generate pcDNA-EGFP. The plasmids expressing EGFP, TRBP, T7, and different mutants of TRBP (TA, TB, and TC) were constructed into pCMV vectors with an N-terminal 3xFLAG epitope digested by HindIII and EcoRI to generate pCMV-3Flag-EGFP, pCMV-3Flag-TRBP, pCMV-3Flag-T7, pCMV-3Flag-TA, pCMV-3Flag-TB, and pCMV-3Flag-TC. The ORFs of EGFP and TRBP were cloned with C-terminal His tag into the SalI and XhoI sites of pDEST-myc-DICER (Addgene, Cat. #19873) to generate pDEST-His-EGFP and pDEST-His-TRBP. The sequence encoding TRBP was cloned into pGEX-4 T-1 digested by BamHI and SalI to generate GST-TRBP. The expression plasmid for human Dicer was purchased from Addgene (Cat. #41584). Human miRNA expression plasmid MIR-21 (pCMV-MIR-21) was purchased from OriGene (Cat. #SC400271). The designed short hairpin RNAs (shRNAs) targeting EGFP or luciferase were cloned into pLKO.5 vector (gift from Dr. Feng Qian) digested by AgeI and EcoRI to produce pLKO-sh-EGFP (shEGFP) and pLKO-sh-LUC (shLUC). The CRISPR/Cas9 plasmids were gifts from Dr. Yongming Wang (Xie et al., 2017). Two designed guide RNAs (gRNAs), gRNA1 and gRNA2, were ligated with tracrRNA-U6 sequence from gRNAU6 plasmid. Then, the fragment was constructed into an epiCRISPR vector that was digested by BspQI to generate epiCRISPR-TRBP. The primer sequences and gRNA sequences are shown in Supplementary Tables 1 and 2.

### Cell Culture Infection and Transfection

293 T cells were seeded in a 6-cm plate at a density of 2 × 10^6^/plate 1 day before infection. Approximately 24 h after inoculation with serum-free DMEM (mock), PR8-WT, or PR8/3841 at a multiplicity of infection (MOI) of 1 as previously described (Li et al., 2016), the infected cells were lysed in TRIzol (Invitrogen) for RNA and protein extraction using the manufacturer’s protocol.

To identify the interaction between NS1 and TRBP, 293 T cells (6 × 10^5^ cells/well) were seeded into a 6-well plate 1 day before transfection. Plasmids expressing NS1 (2 μg) and FLAG-TRBP (2 μg) were co-transfected into 293 T cells using Lipofectamine 2000 (Life Technologies) for 48 h. 293 T cells were transfected with plasmids expressing FLAG-TRBP (2 μg) or FLAG-EGFP (2 μg) for 24 h and then inoculated with PR8-WT (MOI = 1) or WSN-WT (MOI = 1) or PR8/3841 (MOI = 1) in different wells for 24 h.

To determine the effect of the interaction on miRNA production, 293 T cells (6 × 10^5^ cells/well) were seeded into a 6-well plate 1 day before transfection. Plasmids expressing FLAG-TRBP (2 μg) were transfected into TRBP-KO cells using Lipofectamine 2000 for 48 h or TRBP-KO cells were transfected with pCMV-MIR-21 (2 μg) and FLAG-TRBP (2 μg) or FLAG-EGFP (2 μg) for 24 h and then inoculated with DMEM (mock) or PR8-WT (MOI = 1) or PR8/3841 (MOI = 1) in different wells for 24 h.

For the EGFP RNAi assay, 293 T cells (4 × 10^5^ cells/well) were seeded into a 12-well plate one day before transfection. pCMV-3Flag-EGFP (0.1 μg) and shEGFP (0.3 μg) or shLUC (0.3 μg) were co-transfected into cells using Lipofectamine 2000. Six hours post-transfection, the cells were infected with PR8-WT (MOI = 1) or PR8/3841 (MOI = 1). After 48 h post-transfection, cells were washed with PBS and lysed in TRIzol for RNA and protein extraction using the manufacturer’s protocol.

### Co-immunoprecipitation and AGO-Immunoprecipitation

293 T cells transfected with Flag-tagged plasmids or infection with viruses were co-immunoprecipitated (co-IP) by anti-FLAG affinity resin (GenScript). Briefly, 293 T cells lysates in lysis buffer (20 mM Tris–HCl [pH 7.5], 150 mM NaCl, 0.5% NP-40, 5 mM MgCl_2_, 10% glycerol) mixed with a protease inhibitor (Roche) were incubated with 30 μl anti-FLAG affinity resin for 4 h at 4°C in the presence or absence of 10 μg/ml RNase A (Thermo Fisher Scientific) and 5 U/ml RNase III (NEB). After five times washes with 1 × wash buffer (IBA BioTAGnology), the precipitated complexes were used to detect specific proteins by Western blotting.

For AGO-IP, cells lysates in 1 ml RIPA (Cell Signaling Technology) were precleared by incubation with 20 μl of protein A/G PLUS-Agarose (Santa Cruz Biotechnology) and 2 μg of mouse IgG (Santa Cruz Biotechnology) for 1 h at 4°C for pre-clearing. 2 μg of Anti-pan Ago antibody (Millipore) or 2 μg of mouse IgG antibodies (Santa Cruz Biotechnology) and 20 μl of protein A/G PLUS-Agarose were added into the lysates and incubated together for 4 h at 4°C followed by washing five times with 1 × wash buffer. Total RNAs were extracted from the precipitated complexes using TRIzol to construct small RNA libraries.

### Protein Purification and GST Pulldown

Plasmids expressing GST and GST-TRBP were expressed in Escherichia coli BL21 (DE3) strain cells. The cells were harvested and sonicated in lysis buffer (50 mM Tris–HCl [pH 8.0], 50 mM NaCl, 5 mM *β*-mercaptoethanol, 5% glycerol). Then, lysates were cleared by centrifugation at 20,000 *g* for 30 min at 4°C. The supernatants were purified with Glutathione Resin (GenScript) and dialyzed overnight at 4°C. GST and GST-TRBP proteins were detected by SDS-PAGE and used for GST pulldown.

For GST pulldown, the GST and GST-TRBP proteins were bound to glutathione beads and incubated with lysates expressing NS1 or NS1 38/41 protein for 5 h at 4°C in the presence or absence of 10 μg /ml RNase A and 5 U/ml RNase III. After five times washes with 1 × wash buffer, the bound proteins were detected by SDS-PAGE and Western blotting analysis.

### Western and Northern Blotting Assays

Two assays were performed as previously described (Li et al., 2016; Wang et al., 2020). The following primary antibodies were used for detection: *β*-actin (Cell Signaling Technology), TRBP, PACT, Dicer, and EGFP (Santa Cruz Biotechnology), Flag and His (GenScript). The antibody of IAV-NS1 was gift of Dr. Yan Zhou. For Northern blotting, 10 μg of total RNA was used to detect miRNA. The probes used in this study are listed in Supplementary Table 3.

### Generation of KO Cell Lines

293 T cells (1 × 10^6^ cells/well) were seeded into a 6-well plate 1 day before transfection. The CRISPR/Cas9 plasmid (2 μg) were transfected into 293 T cells (80%–90% confluent) with Lipofectamine 2000. 24 h after transfection, cells were selected by puromycin (2.5 μg/ml). 72 h after selection, cells were identified by PCR and Western blotting. For analysis of single cell-derived clones, separated cells were plated at a density of 100–300 cells per 100 cm dish and were incubated for 2 weeks until colony formation. The KO cell lines were confirmed by DNA sequencing and Western blotting.

### RT-qPCR

One microgram of RNA was reverse transcribed to cDNA using HiScript III First Strand cDNA Synthesis kit (+gDNA wiper; Vazyme). qPCR was performed using ChamQ Universal SYBR qPCR Master Mix (Vazyme). All samples were performed in triplicate. The results were normalized to *β*-actin mRNA. The expression levels of specific miRNAs were analyzed by quantitative PCR with specific stem-loop RT primer. The results were normalized to U6 small nuclear RNA. The primer sequences used in RT-qPCR are listed in Supplementary Table 4.

### Animals

BALB/c and C57BL/6 mice were purchased from Charles River Laboratory (Shanghai, China). All animal experiments were carried out under the guidelines of the Institutional Animal Care and Use Committee, Fudan University of China.

### Intranasal Infections

Six- to eight-week-old female BALB/c and C57BL/6 mice were kept under specific pathogen-free conditions in individual ventilated cages. Briefly, mice were anesthetized by intraperitoneal injection of a mixture of atropine, diazepam, and pentobarbital and infected intranasally with 10^4^ PFU PR8/3841 in 50 μl of PBS or 10^4^ PFU PR8-WT in 50 μl of PBS. Total RNAs were extracted from the lung tissues of mice 4 days post-infection (dpi).

### Construction of Small RNA Libraries

RNA extractions were used for the construction of small RNA libraries by the method that depends on the 5′ monophosphate of small RNAs as previously described with the TruSeq Small RNA Sample Preparation Kit of Illumina (San Diego, CA; Li et al., 2016).

### Deep Sequencing and Bioinformatic Analysis of Small RNAs

Libraries of small RNAs were cloned from the RNA samples and sequenced by Illumina HiSeq 2000/2500. Small RNA reads were mapped to the virus genome references or compared to mature miRNAs with a perfect match by Bowtie 1.1.2 before removed from adapter sequences. Bioinformatics analysis of virus-derived small RNAs was conducted using in-house Perl scripts as previously described (Li et al., 2016). Pairs of complementary 22-nt vsiRNAs in each library with different base-pairing lengths were computed using a previously described algorithm (Li et al., 2013). Content and properties of the small RNA libraries sequenced are shown in Supplementary Table 5. The following reference sequences were used in this study:

PR8-WT: The sequences were downloaded from NCBI: AF389115.1, AF389116.1, AF389117.1, AF389118.1, AF389119.1, AF389120.1, AF389121.1, and AF389122.1.

PR8/3841: Obtained from PR8-WT by mutating amino acids R38A and K41A in the NS1 segment.

Mature miRNAs: miRbase 21 (http://www.mirbase.org/).

## Results

### IAV NS1 Interacts With Host TRBP Protein

**Figure 1:**
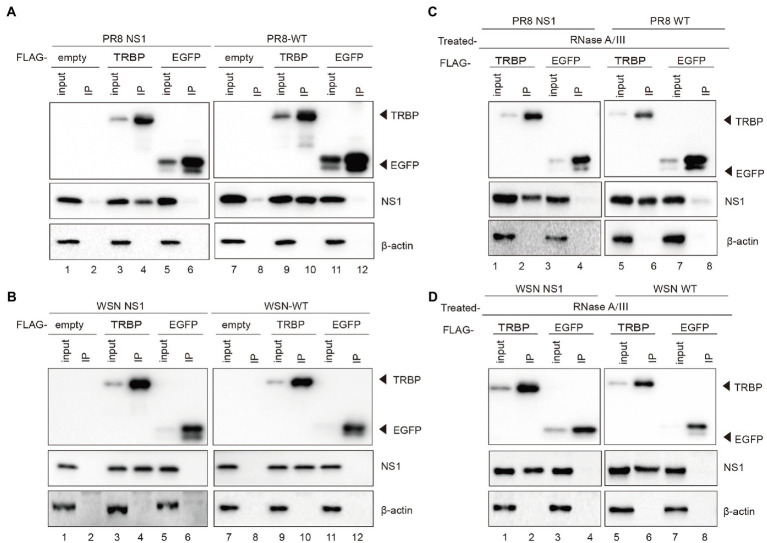
IAV NS1 interacts with TRBP. **(A)** Plasmids encoding PR8 NS1 and FLAG-TRBP or FLAG-EGFP or empty vector were co-transfected into 293 T cells for 48 h (lanes 1–6). 293 T cells were transfected with FLAG-TRBP or FLAG-EGFP or empty vector for 24 h before infected with PR8-WT (MOI = 1) for 24 h (lanes 7–12). Immunoprecipitations were performed with anti-FLAG antibody. FLAG-tagged proteins, NS1, and *β*-actin were detected with specific antibodies. **(B)** Plasmids encoding WSN NS1 and FLAG-TRBP or FLAG-EGFP or empty vector were co-transfected into 293 T cells for 48 h (lanes 1–6). 293 T cells were transfected with FLAG-TRBP or FLAG-EGFP or empty vector for 24 h before infected with WSN-WT (MOI = 1) for 24 h (lanes 7–12). Immunoprecipitations were performed and processed as in **(A)**. **(C,D)** The NS1-specific interaction with TRBP persists in the presence of RNase. RNase A (10 mg/ml) and RNase III (5 U/ml) were treated with cell lysates. Immunoprecipitations were performed with anti-FLAG antibody. Samples were analyzed by SDS–PAGE with the indicated antibodies.

To explore RNAi suppression by the NS1 protein of IAV, we conducted co-immunoprecipitation (co-IP) experiments to identify interactions between IAV NS1 and cellular proteins that are involved in RNAi. We detected NS1 in TRBP immunoprecipitants when plasmids encoding FLAG-tagged TRBP or FLAG-tagged EGFP (negative control) and plasmid encoding PR8 NS1 or WSN NS1 were co-transfected into 293 T cells (Figure 1A, lanes 1–6 and Figure 1B, lanes 1–6). To confirm whether NS1 is associated with TRBP upon IAV infection, 293 T cells were infected with PR8-wildtype (WT) or WSN-WT after ectopically expressing TRBP protein. NS1 was specifically co-immunoprecipitated with TRBP with viral infection, whereas NS1 was undetected in the control of EGFP (Figure 1A, lanes 7–12, Figure 1B, lanes 7–12, and Supplementary Figure 1). Because NS1 and TRBP are both dsRNA binding proteins (dsRBPs), we then examined whether the interaction is dependent on RNA. RNase A and RNase III were added into the cell lysate to exclude potential interactions mediated by single-stranded RNA (ssRNA) and dsRNA (Supplementary Figure 2). The NS1-TRBP interaction was maintained with RNase A and RNase III treatment (Figures 1C,D). These results suggest that the NS1-TRBP interaction is likely mediated by protein–protein binding instead of RNA.

**Figure 2:**
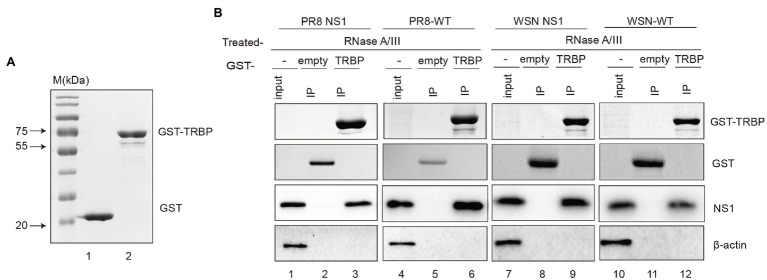
GST pull-down assays of NS1 and TRBP. **(A)** Purification of GST and GST-TRBP proteins. **(B)** GST pull-down assay showing the interaction between NS1 and TRBP. Lysates of 293 T cells transfected with PR8 NS1 or WSN NS1 or infected with PR8-WT (MOI = 1) or WSN-WT (MOI = 1) were incubated with an equal amount of GST or GST-TRBP bound to glutathione-sepharose 4B beads with RNase A (10 mg/ml) and RNase III (5 U/ml) treatment. Samples were analyzed by SDS–PAGE with the indicated antibodies.

To further characterize complex formation, we aimed to validate the interaction using purified NS1 and TRBP. Unfortunately, full-length NS1 protein was observed to aggregate at various concentrations, consistent with those previously reported (Bornholdt and Prasad, 2008; Koliopoulos et al., 2018; Chen et al., 2020b). We finally purified the recombinant glutathione-S-transferase (GST)-fusion TRBP protein and performed GST pull-down experiments. PR8 NS1 or WSN NS1 from ectopic expression or viral infection in 293 T cells was incubated with GST-TRBP or GST purified from E. coli (Figure 2A). We found that NS1 bound with high affinity to GST-TRBP but not to GST with RNase treatment (Figure 2B). Together, these results demonstrate that IAV NS1 physically binds to the host TRBP protein.

### R38A and K41A Mutations in NS1 Abolish the NS1-TRBP Interaction

**Figure 3:**
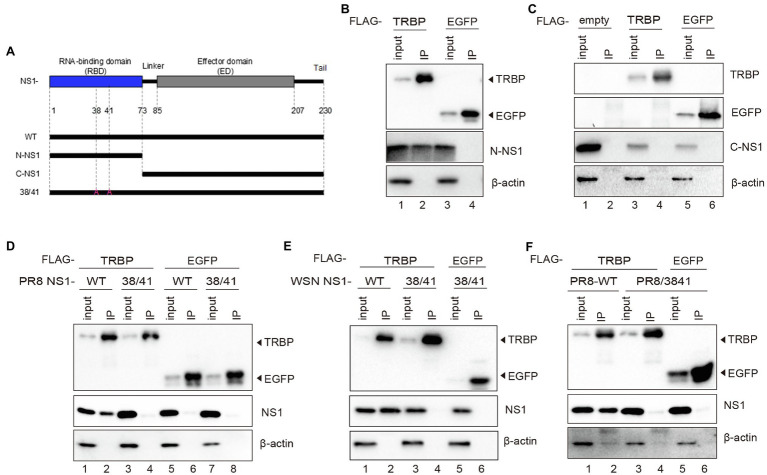
R38A and K41A mutations in NS1 abolish the NS1-TRBP interaction. **(A)** Domain architecture of PR8 NS1 protein (NS1-WT) and the generated mutant constructs (N-NS1, C-NS1, NS1 38/41). **(B)** Immunoprecipitation of N-NS1 protein with the TRBP protein. Plasmids encoding FLAG-TRBP or FLAG-EGFP and N-NS1 were co-transfected into 293 T cells for 48 h. Immunoprecipitations were performed with anti-FLAG antibody. FLAG-tagged proteins, N-NS1, and *β*-actin were detected with specific antibodies. **(C)** Immunoprecipitation of C-NS1 protein with the TRBP protein. Plasmids encoding FLAG-TRBP or FLAG-EGFP and C-NS1 were co-transfected into 293 T cells for 48 h and processed as in **(B)**. **(D)** Plasmids encoding FLAG-TRBP or FLAG-EGFP and PR8 NS1-WT or PR8 NS1 38/41 were co-transfected into 293 T cells for 48 h and processed as in **(B)**. **(E)** Plasmids encoding FLAG-TRBP or FLAG-EGFP and WSN NS1-WT or WSN NS1 38/41 were co-transfected into 293 T cells for 48 h and processed as in **(B)**. **(F)** 293 T cells were transfected with FLAG-TRBP or FLAG-EGFP for 24 h before infected with PR8-WT (MOI = 1) or PR8/3841 (MOI = 1) for 24 h and processed as in **(B)**.

It has been demonstrated that a variety of important sites mediate interactions between NS1 and cellular proteins (Ji-Young Min, 2006; Mibayashi et al., 2007; Hale et al., 2008; Krug, 2015; Moriyama et al., 2016; Schierhorn et al., 2017). To further identify the binding sites in NS1, we constructed the C-terminal deletion mutant of NS1 (N-NS1) and the N-terminal deletion mutant of NS1 (C-NS1; Figure 3A). N-NS1 was specifically co-immunoprecipitated with TRBP (Figure 3B). However, we did not detect the interaction between C-NS1 and TRBP protein (Figure 3C). It was inferred that the N-terminal domain of IAV NS1 harbored critical sites that mediated the interaction with TRBP protein. We thus generated NS1 mutants and found that two-point mutations within NS1 (R38A/K41A) completely abolished NS1-TRBP interaction (Figures 3D,E). Furthermore, a recombinant virus carrying the R38A-K41A substitutions in NS1 (PR8/3841) was rescued to identify the interaction. The results of the virus-infected group were consistent with the plasmid-transfected group (Figure 3F). These results demonstrate that the region comprised of R38 and K41 is essential to bind TRBP protein.

### The First Two Domains of the TRBP Protein Mediate the Interaction With NS1

**Figure 4:**
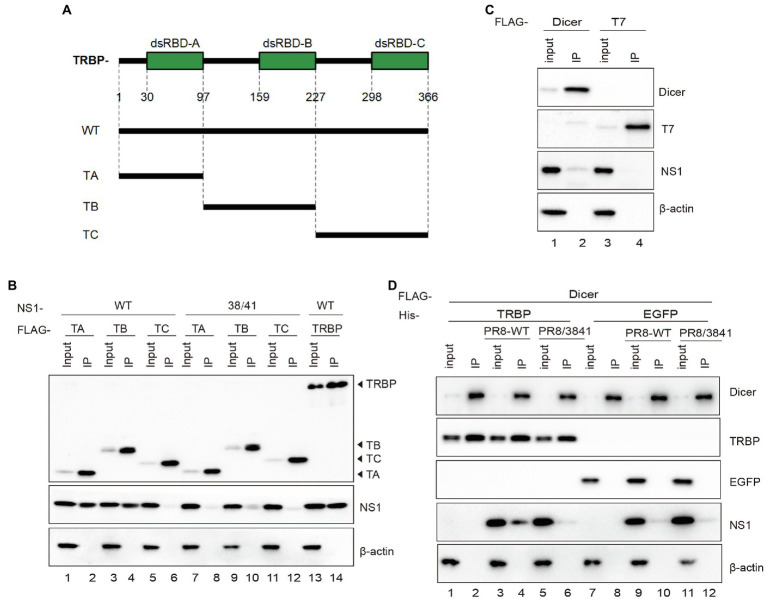
The dsRBD-A and dsRBD-B of TRBP are critical for interacting with NS1. **(A)** Domain architecture of TRBP protein (TRBP-WT) and the generated mutant constructs (TA, TB, and TC). **(B)** Immunoprecipitation of PR8 NS1 protein with different mutants of TRBP proteins. PR8 NS1 and the indicated FLAG-tagged constructs were co-transfected into 293 T cells, followed by FLAG immunoprecipitation. FLAG-tagged proteins, NS1, and *β*-actin were detected with specific antibodies. **(C)** The weak interaction between Dicer and NS1. 293 T cells were transfected with FLAG-Dicer or FLAG-T7 for 24 h before infected with PR8-WT (MOI = 1) for 24 h and processed as in **(B)**. **(D)** NS1 participates in the complex with TRBP and Dicer. 293 T cells were co-transfected with FLAG-Dicer and His-EGFP or His-TRBP for 24 h before infected with mock or PR8-WT (MOI = 1) or PR8/3841 (MOI = 1) and then processed as in **(B)**.

TRBP contains three dsRNA binding domains (dsRBDs), including dsRBD-A, dsRBD-B, and dsRBD-C (Chendrimada et al., 2005; Haase et al., 2005; Kok et al., 2007). The first two domains bind dsRNA, while the third domain mediates protein–protein interactions including the Dicer protein (Daniels et al., 2009). To determine which domain NS1 binds to, we constructed FLAG-tagged plasmids expressing different domains of TRBP (Figure 4A). NS1 and each plasmid encoding TRBP including full-length plasmid (TRBP-WT) and truncated plasmids (TA, TB, and TC) were co-transfected into 293 T cells. Co-IP assays and Western blotting were conducted to identify the interaction region. It showed that the dsRBD-A and dsRBD-B of TRBP were responsible for NS1-TRBP interaction (Figure 4B).

TRBP forms a complex with Dicer and promotes the activity of Dicer in the processing of sRNAs (Chendrimada et al., 2005; Takahashi et al., 2014; Kurzynska-Kokorniak et al., 2015). To confirm the relationship between NS1 and Dicer in viral infection, we expressed FLAG-tagged Dicer or FLAG-tagged T7 (negative control) in 293 T cells and infected with PR8-WT. The results showed a weak interaction between NS1 and Dicer (Figure 4C). We subsequently tested whether the interaction between TRBP and Dicer was influenced by NS1. FLAG-Dicer and His-tagged TRBP or His-tagged EGFP (negative control) were co-transfected into 293 T cells and then infected with mock or PR8-WT or PR8/3841. Co-IP analysis were performed to examine the interaction between Dicer-TRBP and Dicer-NS1. The TRBP-Dicer interaction was not influenced with or without viral infection. However, the NS1-Dicer interaction was enhanced during PR8-WT infection (Figure 4D). We previously found that TRBP interacted with NS1 *via* its RBD instead of the ED that interacted with Dicer. Therefore, we inferred that the NS1 protein might be in the complex with TRBP and Dicer during sRNAs production.

### NS1-TRBP Interaction Reduces the Expression of Endogenous miRNAs

**Figure 5:**
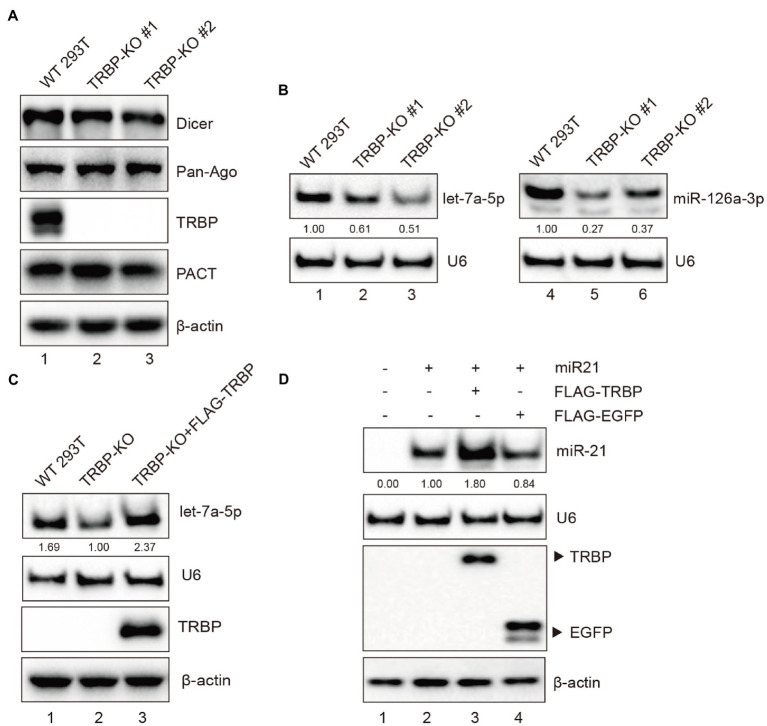
TRBP affects miRNAs production. **(A)** RISC-associated proteins of 293 T and TRBP-KO cells were detected by Western blotting. **(B)** Northern blotting analysis of miR-126a-3p and let-7a-5p expression levels in the indicated cells. The relative expression levels of miRNAs are normalized to that of U6 small nuclear RNA and to the miRNA level from the WT 293 T cells. **(C)** TRBP promotes endogenous let-7a-5p expression. FLAG-TRBP was transfected into TRBP-KO cells for 48 h. Northern blotting and Western blotting were performed to detect let-7a-5p, U6, TRBP, and *β*-actin expressions in the indicated cells. The relative expression level of let-7a-5p is normalized to that of U6 small nuclear RNA and to the miRNA level from the TRBP-KO cells. **(D)** TRBP promotes exogenous miR-21 expression. TRBP-KO cells were transfected with a miR-21 expressing plasmid and FLAG-TRBP or FLAG-EGFP for 48 h. Northern blotting and Western blotting were performed to detect expression levels of miR-21, U6, FLAG-tagged proteins and *β*-actin. The relative expression level of miR-21 is normalized to that of U6 small nuclear RNA and to the miRNA level from the miR-21-transfected TRBP-KO cells.

The effect of TRBP on the expression level of miRNA has remained elusive (Chendrimada et al., 2005; Melo et al., 2009; Ota et al., 2013; Kim et al., 2014). To verify the effect of TRBP on miRNAs, TRBP knockout (KO) 293 T cells were generated with the CRISPR/Cas9 system. Western blot analysis was performed to confirm the depletion of full-length TRBP protein in TRBP-KO cells. The expression of key protein components of the RISC, including Dicer, AGO, and PACT proteins, were similar between parental and TRBP-KO cells (Figure 5A). Northern blot analysis was performed to confirm miRNA production. We identified that the expression levels of let-7a-5p and the abundance of 22-nt miR-126-3p decreased in the absence of TRBP (Figure 5B and Supplementary Figure 3A), which were concordant to the results of a previous study (Chendrimada et al., 2005; Kim et al., 2014).

To validate the function of TRBP, TRBP-KO cells were transfected with FLAG-TRBP plasmid. It showed that TRBP overexpression indeed upregulated let-7a-5p (Figure 5C and Supplementary Figure 3B). We also evaluated the expression level of exogenous miR-21 as previously described (Wang et al., 2020). The human miR-21 expressing plasmid and FLAG-TRBP or FLAG-EGFP (negative control) were co-transfected into TRBP-KO cells. It revealed that TRBP also affected exogenous miR-21 expression (Figure 5D and Supplementary Figure 3C). These results demonstrate that TRBP affects the maturation of miRNAs in 293 T cells.

**Figure 6:**
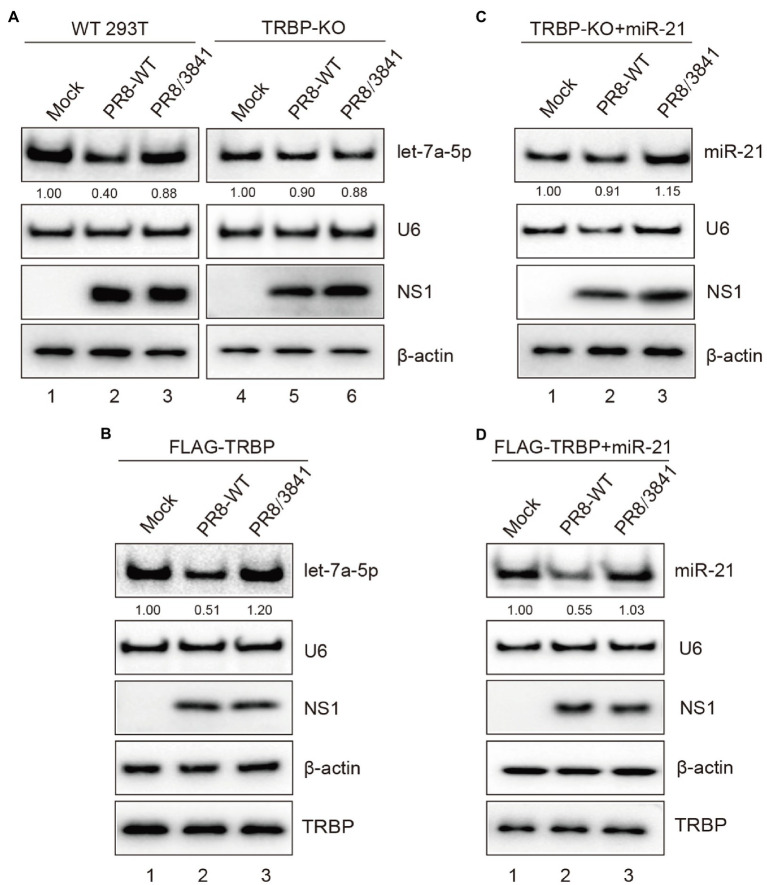
NS1-TRBP interaction reduces miRNAs production. **(A)** The expression level of let-7a-5p is downregulated in PR8-infected WT cells. WT 293 T cells and TRBP-KO cells were infected with mock or PR8-WT (MOI = 1) or PR8/3841 (MOI = 1) for 48 h. Northern blotting and Western blotting were performed to detect expression levels of let-7a-5p, U6, NS1, and *β*-actin. The relative expression level of let-7a-5p is normalized to that of U6 small nuclear RNA and to the miRNA level from the uninfected WT 293 T cells or the uninfected TRBP-KO cells. **(B)** NS1-TRBP interaction reduces the expression of endogenous let-7a-5p. TRBP-KO cells were transfected with FLAG-TRBP for 24 h before infected with PR8-WT or PR8/3841 for 24 h. Northern blotting detection of let-7a-5p and U6 expressions. The relative expression level of let-7a-5p is normalized to that of U6 small nuclear RNA and to the miRNA level from the uninfected cells. Western blotting detection of TRBP, NS1, and *β*-actin proteins. **(C, D)** NS1-TRBP interaction reduces the expression of exogenous miR-21. **(C)** TRBP-KO cells were transfected with a miR-21 expressing plasmid for 24 h before infected with mock or PR8-WT or PR8/3841 for 24 h. **(D)** TRBP-KO cells were co-transfected with FLAG-TRBP and miR-21 expressing plasmid for 24 h before infected with mock or PR8-WT or PR8/3841 for 24 h. Northern blotting detection expressions of miR-21 and U6. The relative expression level of miR-21 is normalized to that of U6 small nuclear RNA and to the miRNA level from the uninfected cells. Western blotting detection of TRBP, NS1, and *β*-actin proteins.

To further elucidate the role of NS1 in miRNA expression mediated by TRBP, we first compared the expression level of the specific miRNA after viral infection of WT and TRBP-KO cells. We found that the abundance of let-7a-5p was significantly reduced in PR8-infected WT cells (Figure 6A, lanes 1–3 and Supplementary Figure 3D). In contrast, infection with or without virus did not alter the expression of let-7a-5p in TRBP-KO cells (Figure 6A, lanes 4–6 and Supplementary Figure 3E). We then transfected FLAG-TRBP in TRBP-KO cells and then infected with mock or PR8-WT or PR8/3841. The same results were observed that PR8-WT instead of PR8/3841 inhibited the production of let-7a-5p (Figure 6B and Supplementary Figure 3F), which suggested that NS1-TRBP interaction affected the production of the endogenous miRNA. NS1 has been shown to be involved in the regulation of miRNAs processing (Terrier et al., 2013; Tan et al., 2014; Bamunuarachchi et al., 2021). To exclude the potential effect of the endogenous miRNA, we also examined the expression of exogenous miR-21. A reduction in miR-21 expression was observed after infection with PR8-WT, while NS1 mutants did not affect miR-21 expression (Figures 6C,D, and Supplementary Figures 3G,H). Taken together, IAV NS1 influences the function of Dicer by binding to the TRBP protein.

### R38A-K41A Substitutions Abolish the Inhibition of shRNA-Mediated RNAi by NS1

**Figure 7:**
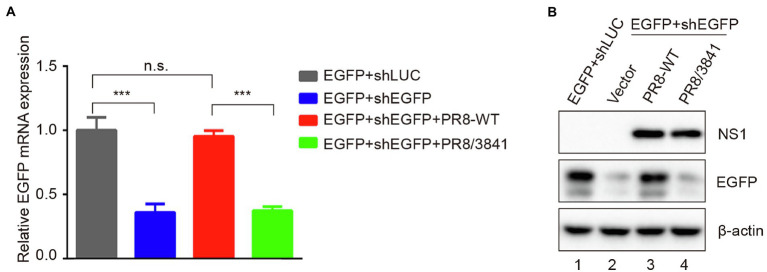
R38A-K41A substitutions in NS1 abolish the inhibition of shRNA-mediated RNAi. **(A)** Relative mRNA levels of EGFP as quantified by qRT-PCR. 293 T cells were co-transfected with a plasmid encoding EGFP and EGFP-specific shRNA (shEGFP) or luciferase-specific shRNA (shLUC) as a negative control and then infected with PR8-WT (MOI = 1) or PR8/3841 (MOI = 1). After 48 h post-transfection, total RNAs were extracted, and the level of EGFP mRNA was examined by qRT-PCR. The experiments were repeated three times independently. The mRNA level of EGFP+shLUC group was set as 1. ***p < 0.001 (Student’s t-test). ns indicates no significance. **(B)** Western blotting detection of EGFP, NS1, and *β*-actin proteins.

Dicer can cleave short hairpin RNA (shRNA) into siRNAs (Haasnoot et al., 2007; Jinek and Doudna, 2009; Qian et al., 2020). As a VSR, NS1 modulates functions of Dicer to facilitate viral replication and pathogenesis (Bucher et al., 2004; Li et al., 2004; de Vries et al., 2009). To further determine whether mutant NS1 was capable of affecting Dicer’s activities, shRNAs were assessed. The EGFP-specific short hairpin RNA (shEGFP) can induce shRNA-mediated silencing to destroy the EGFP transcript. Luciferase-specific shRNA (shLUC) was used as a negative control. We observed that, compared to co-transfection of EGFP with shLuc in 293 T cells, transfection with EGFP and shEGFP statistically significantly reduced EGFP mRNA level (Figure 7A). We then infected 293 T cells with PR8-WT or PR8/3841 after transfection with EGFP and shEGFP. Quantitative real-time PCR (qRT-PCR) and Western blotting analysis showed that the mRNA expression level of EGFP was recovered in PR8-WT-infected cells instead of PR8/3841-infected cells (Figures 7A,B). These results show that infection of PR8-WT effectively suppresses shRNA-induced silencing in 293 T cells. However, PR8/3841 is deficient in the ability to suppress the process.

### Abundant vsiRNAs are Produced *in vitro* and *in vivo* With PR8/3841 Infection

**Figure 8:**
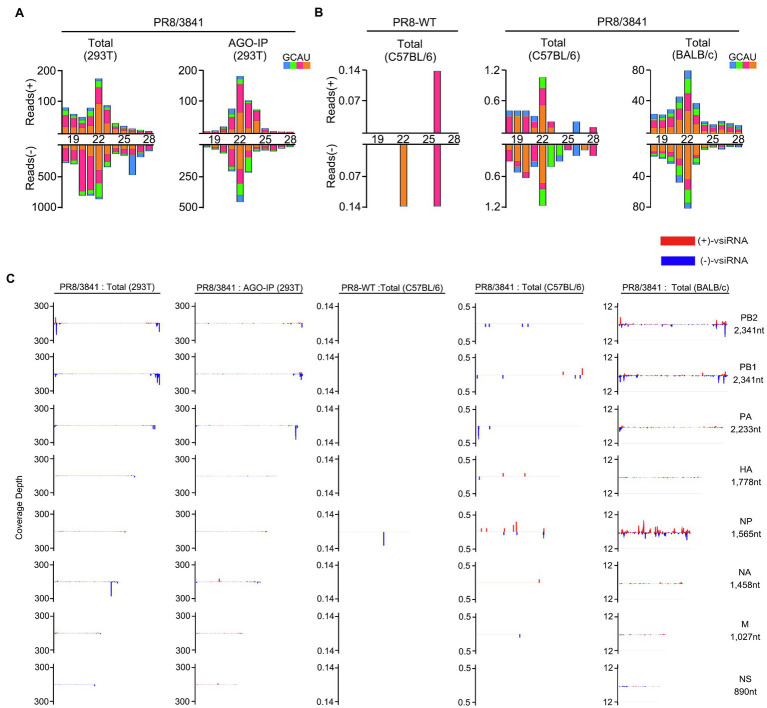
The abundant vsiRNAs produced in mammalian cells and mice with PR8/3841 infection. **(A)** Properties of vsiRNAs (per million total mature miRNAs) sequenced from 293 T cells without or with AGO-IP (at 1 dpi). Size distribution, abundance, the 5′-nucleotide preference of vsiRNAs (per million total 18–28 nt reads) with PR8/3841 infection. **(B)** Properties of vsiRNAs (per million total mature miRNAs) sequenced from adult C57BL/6 or BALB/c mice (at 4 dpi) infected with PR8-WT or PR8/3841. Size distribution, abundance, the 5′-nucleotide preference of vsiRNAs (per million total 18–28 nt reads) with PR8-WT or PR8/3841 infection. **(C)** Relative abundance of 21–23 nt vsiRNA hotspots mapped to PR8-WT or PR8/3841 genomic RNAs, presented from the 3′ end to the 5′ end.

We previously reported that IAV-WT suppressed vsiRNAs production in mammalian cells. We next investigated whether the 38/41 amino acid sites of NS1 were associated with vsiRNAs induction. Total RNAs from PR8/3841 infected 293 T cells without or with AGO-IP were sequenced. We detected abundant vsiRNAs that were predominantly 22-nt in size from viral positive and negative strands (Figure 8A). The vsiRNA reads were mainly derived from the first three segments (PB2, PB1, and PA), and exhibited a discrete distribution pattern (Figure 8C). In conclusion, amino acids 38/41 of NS1 are key sites to induce and suppress vsiRNA production in mammalian somatic cells.

To date, no study on IAV-derived siRNAs *in vivo* has been reported. We sequenced sRNAs from lung tissues of PR8-WT or PR8/3841 infected C57BL/6 and BALB/c mice by intranasal inoculation. PR8-WT did not generate the visible properties vsiRNAs in C57BL/6 mice (Figure 8B). In contrast, it showed a 22-nt predominant size distribution of IAV-specific siRNAs for both viral positive and negative strands in the PR8/3841-infected C57BL/6 and BALB/C mice (Figure 8B). To further identify the distribution of vsiRNAs on the genome, 21–23-nt vsiRNAs were mapped onto the viral genome and exhibited a discrete distribution pattern with high abundance in the segment PB2, PB1, and NP, especially NP (Figure 8C). 46% of the viral reads were derived from the NP segment in PR8/3841-infected BALB/c mice, while the length of NP segment accounted for 11.5% of the length of the viral genome. In contrast, we previously found that most of the vsiRNA reads were mapped to the segment NS in PR8/delNS1-infected 293 T cells (Li et al., 2016), suggesting the distribution of vsiRNAs varied from recombinant virus-expressing NS1 protein with different mutations. Together, our findings show that vsiRNAs are readily detectable *in vitro* and *in vivo* after infection with PR8/3841.

## Discussion

**Figure 9:**
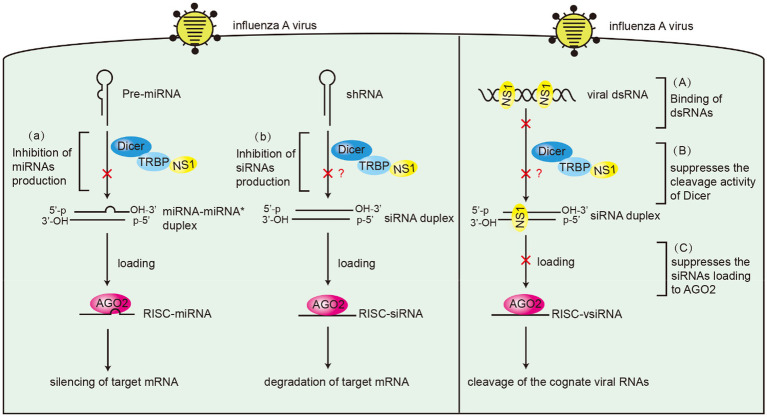
Schematic model of the suppressor roles of NS1 in RNAi. Left panel: **(A)** NS1 inhibits miRNAs production by interacting with TRBP. **(B)** NS1 inhibits shRNA-mediated RNAi by interacting with TRBP. Right panel: **(A)** NS1 inhibits siRNA production by directly binding to dsRNAs. **(B)** NS1 suppresses the cleavage activity of Dicer by interacting with TRBP. **(C)** NS1 suppresses siRNA loading onto AGO2 by directly binding to siRNAs.

Many studies have demonstrated the suppressor role of influenza virus NS1 protein in RNAi. NS1 from different subtypes of influenza viruses suppress RNA silencing *via* dsRBD in Drosophila (Li et al., 2004). NS1 of IAV binds to siRNA and serves as a suppressor of RNA silencing in plants (Bucher et al., 2004). Moreover, we previously reported that NS1 inhibits the generation of vsiRNAs in IAV-infected mammalian cells (Li et al., 2016). An important function of NS1 RBD is binding dsRNA to mediate protein–protein interactions (Chen et al., 2020a). However, not all interactions that occur in RBD are associated with dsRNA. For instance, NS1 directly interacts with importin-α isoforms *via* its amino acids R35, R38, and K41 (Melen et al., 2007). In this study, RNase A and RNase III were used to remove ssRNA and dsRNA. Our results showed that NS1 likely formed an RNA-independent complex with TRBP *in vitro* and TRBP may not compete with dsRNA for binding to NS1. Therefore, as a VSR, NS1 uses various mechanisms for not only dsRNA binding but also interacting with TRBP to interfere with Dicer-mediated sRNA induction (Figure 9). In addition to the role in small RNA processing, TRBP also functions as a PKR inhibitor to suppress the phosphorylation of PKR and eIF2α in interferon response (Park et al., 1994). Besides, NS1 interacts with PKR *via* its 123–127 aa and inhibits translation (Min et al., 2007). The interaction of NS1 and TRBP is likely to affect the activity of PKR indirectly. Future studies should examine whether virus replication is affected by the PKR pathway.

MiRNA expression is influenced by multiple factors. Two factors that we are mainly concerned with include the TRBP protein and viral infection. In this study, we generated 293 T TRBP-KO cells and found that the isoform of miR126-3p and the abundance let-7a-5p were significantly downregulated, which are consistent with the findings of previous studies (Chendrimada et al., 2005; Ota et al., 2013; Kim et al., 2014). There are several reports about the inhibition of IAV in miRNA maturation. Terrier et al. reveal that five miRNAs (miR-21, miR-29a, miR-29b, miR-146a, and miR-452) are downregulated in human lung epithelial cells (A549) infected with H1N1 and H3N2 IAV (Terrier et al., 2013). MiRNA microarray has been used to demonstrate that four miRNAs (miR-221-3p, miR-22-3p, miR-20a-5p, and miR-3,613-3p) are upregulated and two miRNAs (miR-3,178 and miR-4,505) are downregulated in HEK293 cells stably expressing the NS1 protein of H5N1 IAV (Jiao et al., 2019). Another microarray study indicates that 22 and 114 miRNAs in lungs are downregulated at 7 and 15 days post-infection when BALB/c mice are infected with PR8-WT (Tan et al., 2014). However, the mechanism by which the IAV NS1 protein regulates miRNAs is unclear. Here, we compared the effects of miRNA production between NS1 and NS1 38/41 and found that let-7a-5p and miR-21 were downregulated by NS1-TRBP interaction. Based on these results, there are still some issues needed to be resolved in future studies. First, we only tested a few miRNAs, which may not reflect the overall miRNA expression. Second, more types of cells need to be analyzed, preferably the results of *in vivo* IAV infection.

Previous studies have demonstrated that the recombinant viruses containing R38A and K41A mutations in NS1 increase the expression of IFN-*α*/*β* and are attenuated in cells and mice (Talon et al., 2000; Donelan et al., 2003; Ramos et al., 2013). In our study, PR8/3841 induces vsiRNAs following infection, activating the antiviral RNAi *in vivo* and *in vitro*. Dicer-mediated cleavage of viral dsRNAs into vsiRNAs leads to suppression of viral replication by knocking down viral genes. Our recent study indicates that NoV-derived siRNAs have antiviral function *in vivo* by constructing a recombinant SINV containing part of NoV genomic RNA 1 sequences (Zhang et al., 2021). In addition, Zhou and colleagues designed peptides targeting the 3A protein of enterovirus A71 (EV-A71), which abrogate VSR function and promote vsiRNAs production (Fang et al., 2021). These vsiRNAs are able to load into AGO proteins and silence cognate viral RNA *in vivo*. In present work, we show for the first time the production of IAV-derived siRNAs *in vivo*. These IAV vsiRNAs have the characteristics of canonical viral siRNAs and are likely to have antiviral functions. Because both NoV and SINV can infect the muscle tissue of mice, we generated the SINV reporting system to detect the function of vsiRNAs from the NoV virus (Zhang et al., 2021). Unfortunately, because IAV mainly replicates in lung tissues, the SINV reporting system is not suitable for testing the function of IAV vsiRNAs. Future work needs to establish an optimized reporter system to confirm the antiviral function of these IAV siRNAs *in vivo*.

## Data Availability Statement

The datasets presented in this study can be found in online repositories. The names of the repository/repositories and accession number(s) can be found at: https://www.ncbi.nlm.nih.gov/geo/, GSE189776.

## Ethics Statement

The animal study was reviewed and approved by all animal experiments were carried out under the guidelines of the Institutional Animal Care and Use Committee, Fudan University of China.

## Author Contributions

QW and YL performed all infection experiments and wrote the final manuscript. YX performed bioinformatics analyses. JW, ZL, and BW assisted with some experiments. YL conceived the study and designed experiments. All authors contributed to the article and approved the submitted version.

## Funding

This study was supported by grants from the National Natural Science Foundation of China (31770179 and 91640111) and Innovation Program of Shanghai Municipal Education Commission (2017-01-07-00-07-E00015).

## Conflict of Interest

The authors declare that the research was conducted in the absence of any commercial or financial relationships that could be construed as a potential conflict of interest.

## Publisher’s Note

All claims expressed in this article are solely those of the authors and do not necessarily represent those of their affiliated organizations, or those of the publisher, the editors and the reviewers. Any product that may be evaluated in this article, or claim that may be made by its manufacturer, is not guaranteed or endorsed by the publisher.
