# Supplementary material for: The Interaction of Influenza A NS1 and Cellular TRBP Protein Modulates the Function of RNA Interference Machinery
Source: Front Microbiol. 2022 Apr 26;13:859420. doi: 10.3389/fmicb.2022.859420 (PMC9087287; doi:10.3389/fmicb.2022.859420)
Supplement: Supplementary file 1 [file Table_1.DOCX]

Supplementary Material

**Supplementary Figures 1-3 and Supplementary Tables 1-5**


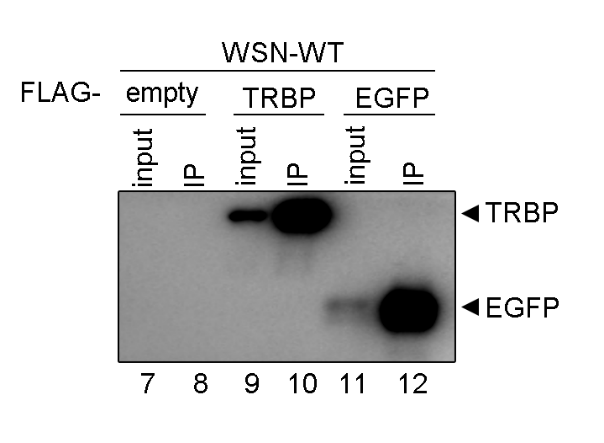


**Supplementary Figure 1.** High exposure of FLAG-tagged proteins corresponding to Figure 1B, lanes 7-12.

**
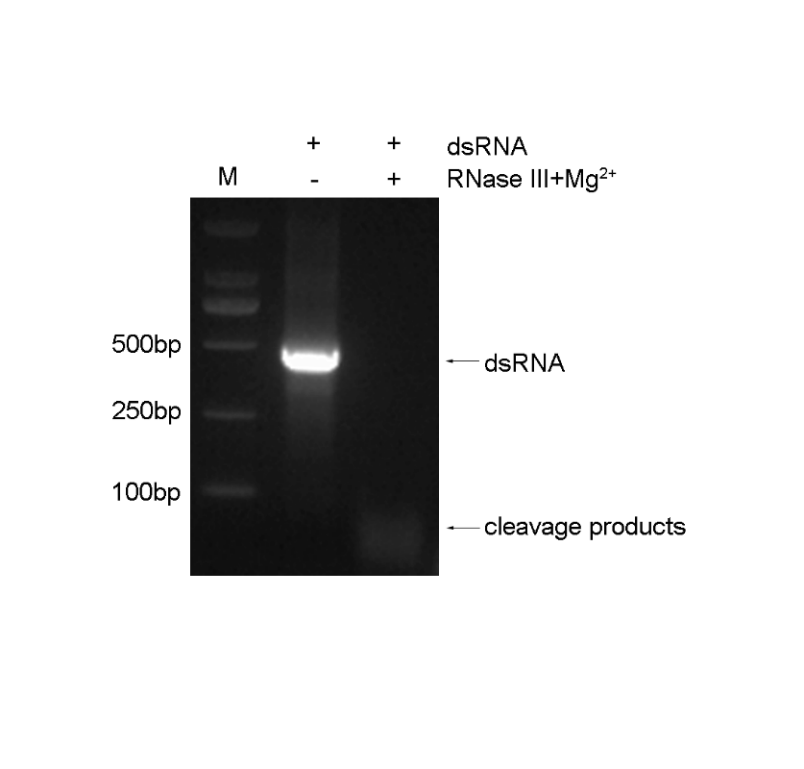
**

**Supplementary Figure 2.** Validation of dsRNA cleavage by RNase III**.** dsRNAs were incubated with or without RNase III+Mg^2+^ in Co-IP lysis buffer. Cleavage products were detected on 3% *agarose* *gel*.


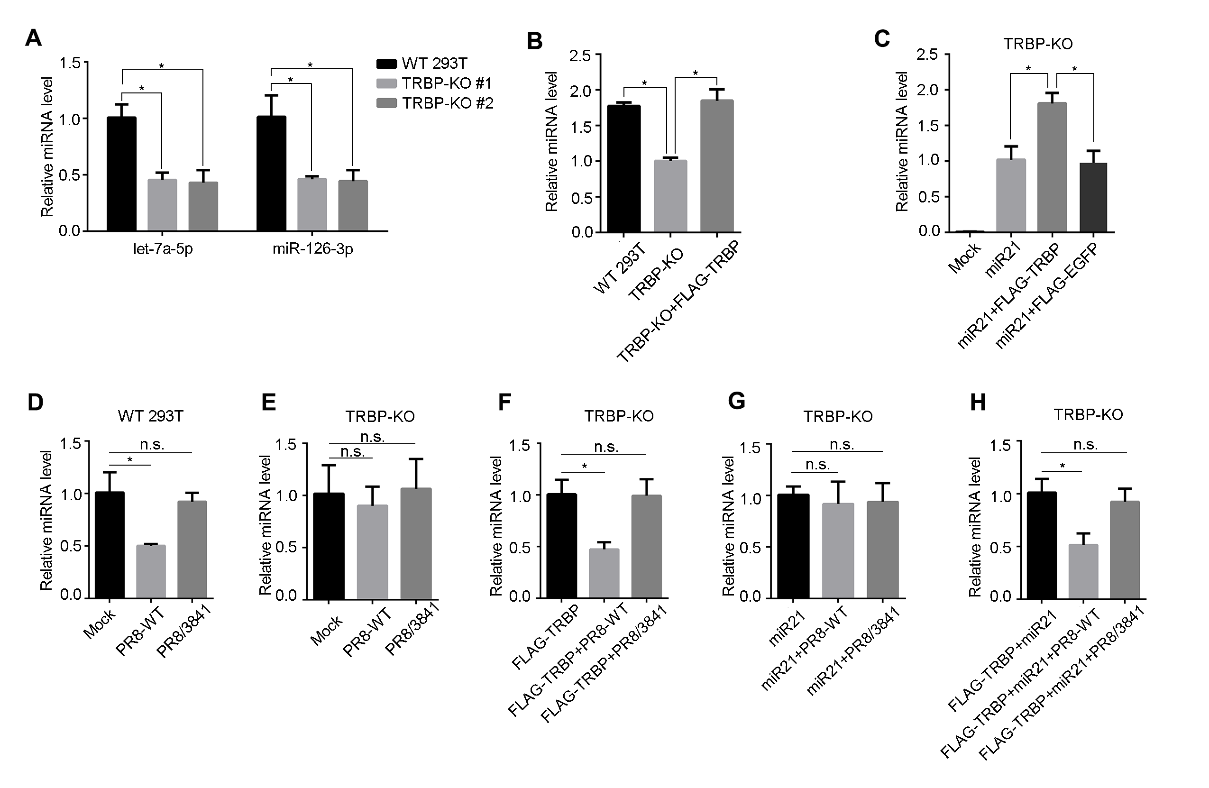


**Supplementary Figure 3.** qRT-PCR measurement of miRNA expressions.

A: qRT-PCR analysis corresponding to Figure 5B.

B: qRT-PCR analysis corresponding to Figure 5C.

C: qRT-PCR analysis corresponding to Figure 5D.

D and E: qRT-PCR analysis corresponding to Figure 6A.

F: qRT-PCR analysis corresponding to Figure 6B.

G: qRT-PCR analysis corresponding to Figure 6C.

H: qRT-PCR analysis corresponding to Figure 6D.

The relative expression level of each miRNA is normalized to that of U6 small nuclear RNA and to the miRNA level from the control cells. Error bars indicate standard deviation of three replicates. Error bars represent SD. * indicates p<0.05. n.s. indicates no significant difference (Student’s t-test).

**Supplementary Table 1.** Primers used for constructing plasmids

| **Primer name** | **Sequence (5’ to 3’)** |
| --- | --- |
| 3Flag-EGFP-F | AAAGACGATGACGACAAGCTTATGGTGAGCAAGGGCGAG |
| 3Flag-EGFP-R | ATCAGATCTATCGATGAATTCTTACTTGTACAGCTCGTC |
| 3Flag-TA-F | AAGACGATGACGACAAGCTTATGAGTGAAGAGGAGCAAG |
| 3Flag-TA-R | TCAGATCTATCGATGAATTCCTACCCCCCTTTGAGGTGTTTG |
| 3Flag-TB-F | AAGACGATGACGACAAGCTTAGCATGCTGGAGCCGGCC |
| 3Flag-TB-R | TCAGATCTATCGATGAATTCCTACACCGTGTGCACTCGAAG |
| 3Flag-TC-F | AAGACGATGACGACAAGCTTCCTCTGGATGCCCGGGATG |
| 3Flag-TC-R | TCAGATCTATCGATGAATTCCTACTTGCTGCCTGCCATG |
| His-EGFP-F | atgggaaccaattcagtcgacATGGTGAGCAAGGGCGAGGAG |
| His-EGFP-R | gctgggtctagatatctcgaggtgatggtgatggtgatgCTTGT |
| shEGFP-F | CCGGGCCACAACGTCTATATCATGGCTCGAGCCATGATATAGACGTTGTGGCTTTTTG |
| shEGFP-R | AATTCAAAAAGCCACAACGTCTATATCATGGCTCGAGCCATGATATAGACGTTGTGGC |
| epiCRISPR-TRBP-F | CGCTCTTCGCCGGTACGACCTTCTCAAAGCCGgttttagagctagaaatagcaa |
| epiCRISPR-TRBP-R | CGCTCTTCTAACGTCGCCAACGGTGACCCGGAcggtgtttcgtcctttccac |
| GST-TRBP-F | GATCTGGTTCCGCGTGGATCCATGAGTGAAGAGGAGCAAG |
| GST-TRBP-R | GATGCGGCCGCTCGAGTCGACTCACTTGCTGCCTGCCATG |

**Supplementary Table 2.** gRNA sequences for constructing the epiCRISPR plasmid

| **gRNA name** | **Sequence (5’ to 3’)** |
| --- | --- |
| TRBP gRNA #1 | GTACGACCTTCTCAAAGCCG |
| TRBP gRNA #2 | GTCGCCAACGGTGACCCGGA |

**Supplementary Table 3.** Probes for Northern blotting

| **Probe name** | **Sequence (5’ to 3’)** |
| --- | --- |
| hsa-let-7a-5p | AACTATACAACCTACTACCTCA |
| hsa-miR126-3p | CGCATTATTACTCACGGTACGA |
| hsa-miR-21 | TCAACATCAGTCTGATAAGCTA |

**Supplementary Table 4.** Primers for RT-qPCR

| **Primer name** | **Sequence (5’ to 3’)** |
| --- | --- |
| Human β-actin mRNA-forward | ACCAACTGGGACGACATGGAGAAA |
| Human β-actin mRNA-reverse | TAGCACAGCCTGGATAGCAACGTA |
| EGFP mRNA-forward | CGTAAACGGCCACAAGTTCA |
| EGFP mRNA-reverse | CTTCATGTGGTCGGGGTAGC |
| miR-21-5p RT-primer | GTCGTATCCAGTGCAGGGTCCGAGGTATTCGCACTGGATACGACT  CAACA |
| let-7a-5p RT-primer | GTCGTATCCAGTGCAGGGTCCGAGGTATTCGCACTGGATACGACA  ACTAT |
| miR-126a-3p RT-primer | GTCGTATCCAGTGCAGGGTCCGAGGTATTCGCACTGGATACGACC  GCATT |
| U6-forward | CTCGCTTCGGCAGCACA |
| U6-reverse | AACGCTTCACGAATTTGCGT |
| miR-21-5p qPCR-forward | GGGTAGCTTATCAGACTGA |
| let-7a-5p qPCR-forward | GGGTGAGGTAGTAGGTTGT |
| miR-126a-3p qPCR-forward | CGTAAACGGCCACAAGTTCA |
| miRNA qPCR-reverse | GTGCAGGGTCCGAGGT |

**Supplementary Table 5.** Content and properties of the small RNA libraries sequenced

| Library | Total reads  (18–28 nt) | miRNA  (mature) | Virus reads  (18–28 nt) | Virus reads of 21–23 nt | | |
| --- | --- | --- | --- | --- | --- | --- |
|  |  |  |  | Reads | % of miRNA | % of all sizes |
| PR8/3841: 293T | 8850668 | 2832376 | 13894 | 6432 | 0.002271 | 0.462934 |
| PR8/3841: 293T AGO-IP  PR8-WT: C57BL/6  PR8/3841: C57BL/6 | 49324664  32139529  32351257 | 17378282  7452281  9760232 | 30993  3  72 | 22171  1  34 | 0.001276  1.34E-07  3.48E-06 | 0.715355  0.333333  0.472222 |
| PR8/3841: BALB/c | 37928939 | 12206465 | 6437 | 3782 | 0.000310 | 0.587541 |
